# Bis(3-hydroxy­pyridinium) fumarate

**DOI:** 10.1107/S1600536809023800

**Published:** 2009-06-24

**Authors:** Lan Shen, Jun-Hua Li, Jing-Jing Nie, Duan-Jun Xu

**Affiliations:** a Department of Obstetrics & Gynecology, Hangzhou Red Cross Hospital, Hangzhou 310003, People’s Republic of China; b Department of Chemistry, Zhejiang University, People’s Republic of China

## Abstract

The crystal structure of the title compound, 2C_5_H_6_NO_2_^+^·C_4_H_2_O_4_^2−^, consists of 3-hydroxy­pyridinium cations and fumarate dianions. The dianion is located on an inversion center and the cation is linked to it by O—H⋯O and N—H⋯O hydrogen bonds. The cation is twisted with respect to the anion by 24.83 (5)°.

## Related literature

For general background, see: Thomas *et al.* (2007 ▶); Fidler *et al.* (2003 ▶); Zhang *et al.* (2004 ▶). For the ionization of hydro­pyridine in the solution, see: Lezina *et al.* (1981 ▶). For 3-hydro­pyridinium salts, see: Aakeroy & Nieuwenhuyzen (1994 ▶); Fukunaga *et al.* (2004 ▶). For co-crystals of neutral pyridine derivatives and neutral fumaric acid, see: Bowes *et al.* (2003 ▶); Aakeroy *et al.* (2002 ▶); Haynes *et al.* (2006 ▶); Bu *et al.* (2007 ▶); Xu *et al.* (2009 ▶). For C—O bond distances in the deprotonated carboxyl groups of fumarates, see: Liu *et al.* (2003 ▶); Liu & Xu (2004 ▶); Xu *et al.* (2009 ▶).
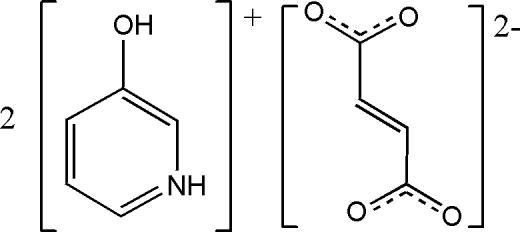


## Experimental

### 

#### Crystal data

2C_5_H_6_NO^+^·C_4_H_2_O_4_^2−^
*M* *_r_* = 306.27
Monoclinic, 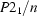

*a* = 3.8037 (5) Å
*b* = 10.4798 (13) Å
*c* = 17.423 (2) Å
β = 90.360 (5)°
*V* = 694.52 (15) Å^3^
*Z* = 2
Mo *K*α radiation
μ = 0.12 mm^−1^
*T* = 294 K
0.32 × 0.28 × 0.24 mm

#### Data collection

Rigaku R-AXIS RAPID IP diffractometer
Absorption correction: none
7561 measured reflections
1359 independent reflections
1237 reflections with *I* > 2σ(*I*)
*R*_int_ = 0.024

#### Refinement

*R*[*F* ^2^ > 2σ(*F* ^2^)] = 0.037
*wR*(*F* ^2^) = 0.100
*S* = 1.07
1359 reflections
107 parameters
2 restraints
H atoms treated by a mixture of independent and constrained refinement
Δρ_max_ = 0.25 e Å^−3^
Δρ_min_ = −0.14 e Å^−3^

### 

Data collection: *PROCESS-AUTO* (Rigaku, 1998 ▶); cell refinement: *PROCESS-AUTO*; data reduction: *CrystalStructure* (Rigaku/MSC, 2002 ▶); program(s) used to solve structure: *SIR92* (Altomare *et al.*, 1993 ▶); program(s) used to refine structure: *SHELXL97* (Sheldrick, 2008 ▶); molecular graphics: *ORTEP-3 for Windows* (Farrugia, 1997 ▶); software used to prepare material for publication: *WinGX* (Farrugia, 1999 ▶).

## Supplementary Material

Crystal structure: contains datablocks I, global. DOI: 10.1107/S1600536809023800/ng2594sup1.cif

Structure factors: contains datablocks I. DOI: 10.1107/S1600536809023800/ng2594Isup2.hkl

Additional supplementary materials:  crystallographic information; 3D view; checkCIF report

## Figures and Tables

**Table 1:** Hydrogen-bond geometry (Å, °)

*D*—H⋯*A*	*D*—H	H⋯*A*	*D*⋯*A*	*D*—H⋯*A*
N1—H1⋯O1	0.893 (12)	1.687 (12)	2.5774 (14)	175.2 (18)
O3—H3*A*⋯O2^i^	0.839 (14)	1.751 (15)	2.5831 (15)	171.5 (16)

Symmetry code: (i) 
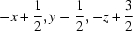
.

**Fig. 1.**
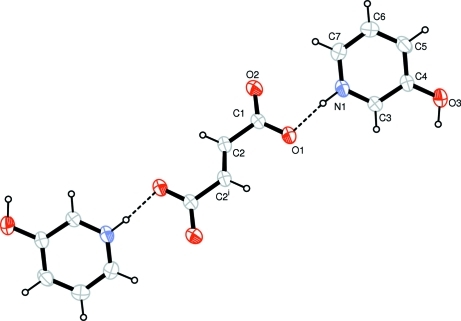
The molecular structure of the title compound with 40% probability displacement (arbitrary spheres for H atoms). Dashed lines indicate hydrogen bonding [symmetry code: (i) 1 - x, 1 - y, 1 - z].

**Fig. 2.**
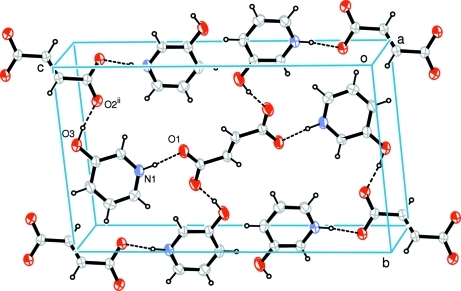
The unit cell packing diagram showing O—H···O and N—H···O hydrogen bonding (dashed lines) [symmetry code: (ii) -x + 1/2, y + 1/2, -z + 1/2].

## supplementary crystallographic information

### Comment

The hydropyridine derivatives and the fumaric acid have been extensively applied in biological and medicine fields (Zhang *et al.*, 2004; Thomas *et al.*, 2007; Fidler *et al.*, 2003). Although the carboxyl group of the fumaric acid is usually deprotonated while the pyridine derivatives are protonated in the solution (Lezina *et al.*, 1981), some crystal structures showed that they also exist as co-crystal of neutral molecules (Bowes *et al.* 2003; Aakeroy *et al.*, 2002; Haynes *et al.* 2006; Xu *et al.* 2009). Herein we report the crystal structure of the title compound containing pyridine derivative and fumaric acid components.

The crystal structure of the title compond consists of fumarate anions and 3-hydroxypyridinium cations (Fig. 1). The planar fumarate anion is located in an inversion center. The C1—O1 bond distance of 1.2603 (15) Å is similar to C1—O2 bond distance of 1.2452 (15) Å, it agrees with those found in metal complexes of fumarate (Liu *et al.* 2003; Liu & Xu, 2004).

The 3-hydroxypyridine is protonated in the crystal structure, the geometry data is consistent with those in crystal structures of 3-hydroxypyridinium hydrogen *L*-malate (Aakeroy & Nieuwenhuyzen, 1994) and 3-hydroxypyridinium hydrogen tartronate (Fukunaga *et al.* 2004).

In the crystal structure the planar hydroxypyridinium cation is twisted respect to the planar fumarate with a dihedral angle of 24.83 (5)°, and links with the fumarate anions *via* N—H···O and O—H···O hydrogen bonding (Table 1 and Fig. 2).

### Experimental

Reagents and solvent were used as purchased without further purification. 3-Hydroxypyridine (2 mmol) and fumaric acid (1 mmol) were dissolved in ethanol (5 ml) at room temperature. The single crystals were obtained from the solution after one week.

### Refinement

H atoms bonded to N and O atoms were located in a difference Fourier map and were refined with distance restraints of O—H = 0.82±0.01 and N—H = 0.86±0.01 Å; *U*_iso_(H) = 1.5*U*_eq_(N,*O*). Other H atoms were placed in calculated positions with C—H = 0.93 Å and refined in riding mode with *U*_iso_(H) = 1.2*U*_eq_(C).

### Crystal data

**Table:**

2C_5_H_6_NO^+^·C_4_H_2_O_4_^2^^−^	*F*(000) = 320
*M**_r_* = 306.27	*D*_x_ = 1.465 Mg m^−^^3^
Monoclinic, *P*2_1_/*n*	Mo *K*α radiation, λ = 0.71073 Å
Hall symbol: -P 2yn	Cell parameters from 2322 reflections
*a* = 3.8037 (5) Å	θ = 2.4–24.6°
*b* = 10.4798 (13) Å	µ = 0.12 mm^−^^1^
*c* = 17.423 (2) Å	*T* = 294 K
β = 90.360 (5)°	Prism, colorless
*V* = 694.52 (15) Å^3^	0.32 × 0.28 × 0.24 mm
*Z* = 2	

### Data collection

**Table:**

Rigaku R-AXIS RAPID IP diffractometer	1237 reflections with *I* > 2σ(*I*)
Radiation source: fine-focus sealed tube	*R*_int_ = 0.024
graphite	θ_max_ = 26.0°, θ_min_ = 2.3°
ω scans	*h* = −4→4
7561 measured reflections	*k* = −12→12
1359 independent reflections	*l* = −20→21

### Refinement

**Table:**

Refinement on *F*^2^	Secondary atom site location: difference Fourier map
Least-squares matrix: full	Hydrogen site location: inferred from neighbouring sites
*R*[*F*^2^ > 2σ(*F*^2^)] = 0.037	H atoms treated by a mixture of independent and constrained refinement
*wR*(*F*^2^) = 0.100	*w* = 1/[σ^2^(*F*_o_^2^) + (0.0527*P*)^2^ + 0.1474*P*] where *P* = (*F*_o_^2^ + 2*F*_c_^2^)/3
*S* = 1.07	(Δ/σ)_max_ = 0.001
1359 reflections	Δρ_max_ = 0.25 e Å^−^^3^
107 parameters	Δρ_min_ = −0.14 e Å^−^^3^
2 restraints	Extinction correction: *SHELXL97* (Sheldrick, 2008), Fc^*^=kFc[1+0.001xFc^2^λ^3^/sin(2θ)]^-1/4^
Primary atom site location: structure-invariant direct methods	Extinction coefficient: 0.116 (10)

### Special details

**Table:**

Geometry. All e.s.d.'s (except the e.s.d. in the dihedral angle between two l.s. planes) are estimated using the full covariance matrix. The cell e.s.d.'s are taken into account individually in the estimation of e.s.d.'s in distances, angles and torsion angles; correlations between e.s.d.'s in cell parameters are only used when they are defined by crystal symmetry. An approximate (isotropic) treatment of cell e.s.d.'s is used for estimating e.s.d.'s involving l.s. planes.
Refinement. Refinement of *F*^2^ against ALL reflections. The weighted *R*-factor *wR* and goodness of fit *S* are based on *F*^2^, conventional *R*-factors *R* are based on *F*, with *F* set to zero for negative *F*^2^. The threshold expression of *F*^2^ > σ(*F*^2^) is used only for calculating *R*-factors(gt) *etc*. and is not relevant to the choice of reflections for refinement. *R*-factors based on *F*^2^ are statistically about twice as large as those based on *F*, and *R*- factors based on ALL data will be even larger.

### Fractional atomic coordinates and isotropic or equivalent isotropic displacement parameters (Å^2^)

**Table:**

	*x*	*y*	*z*	*U*_iso_*/*U*_eq_	
N1	0.0136 (3)	0.56088 (11)	0.76518 (6)	0.0373 (3)	
O1	0.2604 (3)	0.49000 (9)	0.63451 (5)	0.0489 (3)	
O2	0.4289 (3)	0.69067 (9)	0.62330 (5)	0.0568 (4)	
O3	−0.2084 (3)	0.39098 (10)	0.93748 (6)	0.0559 (3)	
C1	0.3982 (3)	0.58033 (12)	0.59760 (7)	0.0368 (3)	
C2	0.5267 (3)	0.55371 (13)	0.51822 (7)	0.0375 (3)	
H2	0.6522	0.6174	0.4933	0.045*	
C3	−0.0215 (3)	0.46506 (12)	0.81483 (7)	0.0357 (3)	
H3	0.0526	0.3836	0.8012	0.043*	
C4	−0.1676 (3)	0.48541 (12)	0.88672 (7)	0.0366 (3)	
C5	−0.2754 (3)	0.60885 (13)	0.90499 (7)	0.0410 (3)	
H5	−0.3763	0.6256	0.9524	0.049*	
C6	−0.2323 (4)	0.70583 (13)	0.85271 (8)	0.0431 (3)	
H6	−0.3019	0.7885	0.8647	0.052*	
C7	−0.0849 (4)	0.67954 (13)	0.78221 (8)	0.0418 (3)	
H7	−0.0543	0.7447	0.7466	0.050*	
H1	0.103 (4)	0.5407 (17)	0.7195 (6)	0.063*	
H3A	−0.127 (5)	0.3217 (12)	0.9213 (10)	0.063*	

### Atomic displacement parameters (Å^2^)

**Table:**

	*U*^11^	*U*^22^	*U*^33^	*U*^12^	*U*^13^	*U*^23^
N1	0.0428 (6)	0.0421 (6)	0.0271 (5)	−0.0016 (5)	0.0072 (4)	0.0009 (4)
O1	0.0743 (7)	0.0392 (6)	0.0333 (5)	−0.0081 (4)	0.0207 (5)	−0.0027 (4)
O2	0.0928 (9)	0.0383 (6)	0.0397 (6)	−0.0108 (5)	0.0225 (5)	−0.0095 (4)
O3	0.0852 (8)	0.0450 (6)	0.0378 (6)	0.0079 (5)	0.0253 (5)	0.0091 (4)
C1	0.0467 (7)	0.0355 (7)	0.0283 (6)	−0.0003 (5)	0.0071 (5)	−0.0027 (5)
C2	0.0468 (7)	0.0369 (7)	0.0290 (6)	−0.0030 (5)	0.0103 (5)	0.0003 (5)
C3	0.0420 (7)	0.0342 (6)	0.0310 (6)	0.0005 (5)	0.0076 (5)	−0.0018 (5)
C4	0.0407 (7)	0.0406 (7)	0.0285 (6)	−0.0012 (5)	0.0072 (5)	0.0016 (5)
C5	0.0441 (7)	0.0472 (8)	0.0318 (6)	0.0042 (6)	0.0087 (5)	−0.0056 (6)
C6	0.0481 (7)	0.0363 (7)	0.0450 (7)	0.0055 (5)	0.0039 (6)	−0.0043 (5)
C7	0.0482 (8)	0.0386 (7)	0.0387 (7)	−0.0001 (5)	0.0044 (5)	0.0067 (5)

### Geometric parameters (Å, °)

**Table:**

N1—C7	1.3327 (17)	C2—H2	0.9300
N1—C3	1.3327 (16)	C3—C4	1.3898 (17)
N1—H1	0.893 (12)	C3—H3	0.9300
O1—C1	1.2603 (15)	C4—C5	1.3945 (18)
O2—C1	1.2452 (15)	C5—C6	1.3752 (19)
O3—C4	1.3369 (15)	C5—H5	0.9300
O3—H3A	0.839 (14)	C6—C7	1.3813 (19)
C1—C2	1.4962 (16)	C6—H6	0.9300
C2—C2^i^	1.308 (3)	C7—H7	0.9300
			
C7—N1—C3	121.94 (11)	O3—C4—C3	122.09 (12)
C7—N1—H1	121.9 (12)	O3—C4—C5	120.01 (11)
C3—N1—H1	116.2 (12)	C3—C4—C5	117.90 (11)
C4—O3—H3A	111.9 (13)	C6—C5—C4	119.86 (11)
O2—C1—O1	123.54 (11)	C6—C5—H5	120.1
O2—C1—C2	118.36 (11)	C4—C5—H5	120.1
O1—C1—C2	118.10 (11)	C5—C6—C7	119.49 (12)
C2^i^—C2—C1	123.96 (15)	C5—C6—H6	120.3
C2^i^—C2—H2	118.0	C7—C6—H6	120.3
C1—C2—H2	118.0	N1—C7—C6	120.03 (12)
N1—C3—C4	120.77 (12)	N1—C7—H7	120.0
N1—C3—H3	119.6	C6—C7—H7	120.0
C4—C3—H3	119.6		

Symmetry codes: (i) −*x*+1, −*y*+1, −*z*+1.

### Hydrogen-bond geometry (Å, °)

**Table:**

*D*—H···*A*	*D*—H	H···*A*	*D*···*A*	*D*—H···*A*
N1—H1···O1	0.89 (1)	1.69 (1)	2.5774 (14)	175 (2)
O3—H3A···O2^ii^	0.84 (1)	1.75 (2)	2.5831 (15)	172 (2)

Symmetry codes: (ii) −*x*+1/2, *y*−1/2, −*z*+3/2.
